# Obesity and type 2 diabetes have additive effects on left ventricular remodelling in normotensive patients-a cross sectional study

**DOI:** 10.1186/s12933-017-0504-z

**Published:** 2017-02-08

**Authors:** Kirstie A. De Jong, Juliane K. Czeczor, Smithamol Sithara, Kevin McEwen, Gary D. Loopaschuk, Alan Appelbe, Kimberly Cukier, Mark Kotowicz, Sean L. McGee

**Affiliations:** 1 Metabolic Research Unit, Metabolic Reprogramming Laboratory, School of Medicine, Deakin University, Waurn Ponds, VIC Australia; 2 Institute for Clinical Diabetology, German Diabetes Center, Leibniz Center for Diabetes Research, Heinrich-Heine University, c/o Auf’m Hennekamp 65, 40225 Düsseldorf, Germany; 3 German Center of Diabetes Research, Ingolstädter Landstraße 1, 85764 München-Neuherberg, Germany; 4 Department of Pediatrics, University of Alberta, Edmonton, AB T6G 2H7 Canada; 5 Department of Pharmacology, University of Alberta, Edmonton, AB T6G 2H7 Canada; 6 Cardiology Department, Barwon Health, University Hospital Geelong, Victoria, Australia; 7 Geelong Endocrinology and Diabetes Centre, Geelong, VIC Australia; 8 Endocrinology Department, Barwon Health, University Hospital, Geelong, VIC Australia; 9 School of Medicine, Deakin University, Waurn Ponds, VIC Australia; 10 Melbourne Medical School-Western Precinct, The University of Melbourne, Victoria, Australia

**Keywords:** Obesity, Type 2 diabetes, Left ventricular hypertrophy, Left ventricular diastolic dysfunction, Echocardiography

## Abstract

**Background:**

It is unclear whether obesity and type 2 diabetes (T2D), either alone or in combination, induce left ventricular hypertrophy (LVH) independent of hypertension. In the current study, we provide clarity on this issue by rigorously analysing patient left ventricular (LV) structure via clinical indices and via LV geometric patterns (more commonly used in research settings). Importantly, our sample consisted of hypertensive patients that are routinely screened for LVH via echocardiography and normotensive patients that would normally be deemed low risk with no further action required.

**Methods:**

This cross sectional study comprised a total of 353 Caucasian patients, grouped based on diagnosis of obesity, T2D and hypertension, with normotensive obese patients further separated based on metabolic health. Basic metabolic parameters were collected and LV structure and function were assessed via transthoracic echocardiography. Multivariable logistic and linear regression analyses were used to identify predictors of LVH and diastolic dysfunction.

**Results:**

Metabolically healthy normotensive obese patients exhibited relatively low risk of LVH. However, normotensive metabolically non-healthy obese, T2D and obese/T2D patients all presented with reduced normal LV geometry that coincided with increased LV concentric remodelling. Furthermore, normotensive patients presenting with both obesity and T2D had a higher incidence of concentric hypertrophy and grade 3 diastolic dysfunction than normotensive patients with either condition alone, indicating an additive effect of obesity and T2D. Alarmingly these alterations were at a comparable prevalence to that observed in hypertensive patients. Interestingly, assessment of LVPWd, a traditional index of LVH, underestimated the presence of LV concentric remodelling. The implications for which were demonstrated by concentric remodelling and concentric hypertrophy strongly associating with grade 1 and 3 diastolic dysfunction respectively, independent of sex, age and BMI. Finally, pulse pressure was identified as a strong predictor of LV remodelling within normotensive patients.

**Conclusions:**

These findings show that metabolically non-healthy obese, T2D and obese/T2D patients can develop LVH independent of hypertension. Furthermore, that LVPWd may underestimate LV remodelling in these patient groups and that pulse pressure can be used as convenient predictor of hypertrophy status.

**Electronic supplementary material:**

The online version of this article (doi:10.1186/s12933-017-0504-z) contains supplementary material, which is available to authorized users.

## Background

Obesity and T2D are well-accepted risk factors for the development of left ventricular hypertrophy (LVH) [1, 2]. However, it remains unclear as to whether these stresses are sufficient to cause LVH, independent of hypertension and other cardiac disease. This is largely due to the asymptomatic nature of LVH and the way in which risk is monitored in normotensive obese and/or T2D patients.

LVH is traditionally characterised by increased thickness of the LV posterior wall diameter (LVPWd) and/or increased LV mass [3]. There are three LV geometric patterns that can identify the type of LVH present, eccentric hypertrophy (increased LV mass), concentric remodelling [increased relative wall thickness (RWT) of the LVPWd, normal LV mass] and concentric hypertrophy (increased LV mass and increased RWT). These changes in cardiac structure are often accompanied by diastolic dysfunction (DD, impaired LV relaxation) and can be detected in obese patients via transthoracic echocardiography (TTE) [3]. However, requests for patients to undergo TTE are often restricted to those with chronic hypertension, or to those who have returned an abnormal electrocardiogram result after presenting with symptoms such as arrhythmia, shortness of breath or chest pain. Monitoring risk in obese and/or T2D patients this way presumes that without hypertension, obesity and T2D are insufficient stresses to induce LVH.

Major limitations of past studies are that obesity and T2D have often been considered one disease state, and the individual contributions of these diseases to LVH in the absence of hypertension have remained obscured. Furthermore, in studies that have appropriately distinguished obesity [4, 5] and T2D [6–8], there has been a propensity to use LV mass alone as an independent variable to detect LVH, without assessing the RWT of the LV posterior wall. This restricts the type of LVH that can be detected to eccentric hypertrophy and prevents the detection of concentric remodelling and concentric hypertrophy. Previous studies suggest there is prognostic value in assessment of LV geometric patterns, with increased LV mass and concentric hypertrophy in particular [9, 10] found to associate with increased risk of adverse cardiovascular events. Whether this prognostic value applies to normotensive patients with obesity and T2D remains unresolved.

The current study aimed to determine whether patients presenting with obesity and type 2 diabetes, either alone or in combination, exhibit LVH in the absence of hypertension. Both traditional indices of LVH and LV geometric patterns were used to identify LVH. Furthermore, we sought to determine whether LV geometry predicted the presence of diastolic dysfunction and whether routine metabolic parameters can be used to predict LVH in these patients.

## Methods

### Study approval

This cross sectional study comprised a total of 353 Caucasian patients, from the University Hospital Geelong and the Geelong Endocrinology and Diabetes Centre. The study was conducted in accordance with National Health and Medical Research Council (NHMRC) guidelines and was approved by the Human Research Ethics Committee (HREC) via the Barwon Health Research and Integrity Unit, in accordance to guidelines outlined in section 5 of the National Statement on Ethical Conduct in Human Research.

### Participant groups

Based on the diagnosis of obesity, T2D and hypertension, patients were designated into one of the following groups; normotensive obese (N.Obese) n = 58, normotensive T2D (N.T2D) n = 41, normotensive obese/T2D (N.Obese/T2D) n = 42, hypertensive obese (H.Obese) n = 71, hypertensive T2D (H.T2D) n = 74 and hypertensive obese/T2D (H.Obese/T2D) n = 67. For the diagnosis of obesity, T2D and hypertension, basic clinical and metabolic data were collected for each participant consisting of age, sex, height, weight, blood pressure, HbA1c %, fasting glucose, LDL-C, HLD-C, cholesterol and triglyceride levels (after overnight, 8 h minimum fast), any history of anti-hyperglycaemic or anti-hypertensive medication, and any history of cardiovascular and/or systemic disease.

### Characterisation of and inclusion/exclusion criteria for participant groups

Obesity was characterised as a BMI ≥30 kg/m^2^. Patients with a history of anti-hyperglycaemic medication were permitted within the obese group, due to the increasingly common use of biguanides in pre-diabetic/obese patients. T2D was characterised as having three or more elevated fasting glucose levels within a 12-month period, of ≥7 mmol/l, with or without a history of anti-hyperglycaemic medication. Hypertension was characterised as having both elevated diastolic and systolic blood pressure of ≥140/90 mmHg, with or without a history of anti-hypertensive medication. Those patients with controlled hypertension (i.e. history of hypertension or use of anti-hypertensive medication, with a blood pressure <140/90) were excluded from the study. Additional exclusion criteria for patients of all groups included history of cardiac disease or systemic disease, age <18 years, or the collection of accompanying clinical and metabolic medical records within a time period >6 months before or after TTE procedure. For a more detailed description of the study design see Additional file 1.

### Blood pressure measurements

Blood pressure (BP) was recorded as per the American Heart Association guidelines, with the use of OMRON *Intelli* sense HEM-907 or HBF-1300 and cuff bladder at least 80% of the patient’s arm circumference. In the incidence of an elevated BP reading (≥140/90 mmHg), the measurement was repeated up to three times. With the lowest BP measurement recorded. Pulse pressure mmHg was calculated by subtracting diastolic BP from systolic BP (systolic BP mmHg–diastolic BP mmHg).

### Metabolically healthy vs metabolically non-healthy patients

To separate normotensive obese patients based on metabolic health. We adhered to Karelis criteria. With metabolically healthy patients determined as; fasting glucose ≤5.5 mmol/l, HDL-C ≥1.4 mmol/l, LDL-C ≤2.6 mmol/l, cholesterol ≤5.5 mmol/l and triglycerides ≤1.8 mmol/l. Patients were categorised as being metabolically unhealthy if they exhibited >1 more parameter outside these normal ranges.

### Transthoracic echocardiography

Sonographers were qualified with a Diploma of Medial Ultrasonography or equivalent. Both the sonographers that performed the echocardiography and cardiologists that analysed the results were blinded to the study groups, due to the retrospective nature of the study. All echocardiograms were performed using the Phillips Ie33 with a S5-1 transducer. A combination of two dimensional, M-mode, pulsed wave and continuous wave Doppler and tissue Doppler were used. Left ventricular diameter and wall thicknesses were measured in the parasternal long axis view using two-dimensional or M-mode measurements [left ventricular internal diastolic dimension (LVIDd), left ventricular internal systolic dimension (LVISd), interventricular septum dimension (IVSd), left ventricular posterior wall dimension (LVPWd)]. Of note, while M-mode was used to measure the LV wall thickness whenever possible, in cases where the M-mode was not able to be properly aligned (orthogonal) two dimensional echocardiography was used. Mitral inflow velocities (E’ velocity, Peak E-wave, Peak A-Wave) and deceleration times (DT) were measured using pulsed wave Doppler in the apical 4 chamber view. Echocardiographic data was analysed using proprietary software.

### Characterisation of diastolic dysfunction

Diastolic dysfunction (DD) was characterised according to the American Society of Echocardiography (ASE) guidelines [11]. Patients were graded with either normal diastolic function (E′ ≥ 10 cm/s) or DD, characterised as Grade 1 (impaired relaxation) E′ < 10 cm/s, E/A < 0.8, E/E′ ≤ 8; Grade 2 (pseudonormal) E′ < 10 cm/s, E/A 0.8–1.5, E/E′ 9–14; or Grade 3 (restrictive) E′ < 10 cm/s, E/A ≥ 2, E/E′ > 14.

### Left ventricular geometry

LV mass was estimated according to ASE guidelines [12], in which LV mass (grams) = (0.8·[1.04·(LVEDd + IVSd + LVPWd)^3^ − (LVEDd)^3^]) + 0.6). LV mass was then indexed to body surface area (BSA, g/m^2^) and to height (g/m^2.7^). RWT was calculated using the formula, RWT = ((IVSd + LVPWd)/LVEDd) and via ((2·LVPWd)/LVEDd). LV geometry was characterised using the following criteria; Normal LV geometry, RWT ≤ 42, LVMI (g/m^2.7^) ≤51; eccentric hypertrophy (EH), RWT ≤ 42, LVMI (g/m^2.7^) >51; concentric remodelling (CR), RWT > 42, LVMI (g/m^2.7^) ≤51 and concentric hypertrophy (CH), RWT > 42, LVMI (g/m^2.7^) >51.

### Statistical analysis

Continuous variables were represented as means  ±1 standard deviation (SD), unless otherwise stated. Means of continuous variables were analyzed via ANOVA assessed with Bonferroni, and associations were determined by performing linear regression analysis, assessed with Pearson’s correlation coefficient. Categorical variables were expressed as percentages or prevalence and analyzed via Chi square tests, using fisher’s exact test. To determine independent predictors of categorical variables, multivariable logistic regression analysis was performed with variables adjusted for as detailed. Of note, aortic stenosis was not adjusted for as the presence of aortic stenosis (characterised by an ascending aorta of <3.7 cm) did not associate with diastolic dysfunction or LV geometric patterns in the normotensive patients. p < 0.05 was considered significant. All statistical analysis were performed using SPSS version 23.

## Results

### Basic clinical data

Basic clinical data are presented in Table 1 and Additional file 1: Table S1. Of note, normotensive and hypertensive patients within the same group were of similar age and gender percentage (Table 1). Between groups, age was lower in both T2D and Obese/T2D groups compared to obese groups (p < 0.001), age was however adjusted for in future analysis as detailed.

**Table 1:** Basic clinical data

Group	Obese		T2D		Obese/T2D	
Condition	Norm.	Hyper.	Norm.	Hyper.	Norm.	Hyper.
Basic clinical data						
*n*	58	71	41	74	42	67
Age (years)	48 ± 2.0	54 ± 1.6	68 ± 1.9^ǂǂǂ^	68 ± 1.0^ǂǂǂ^	60 ± 1.9^ǂǂǂ^	65 ± 1.2^ǂǂǂ^
Female %	59	52	40	43	53	48
BMI (kg/m^2^)	34 ± 0.77	37 ± .74	26 ± .40^ǂǂǂ^	26 ± 0.47^ǂǂǂ^	36 ± .71^×××^	36 ± 0.74^×××^
Height (cm)	166 ± 1.4	167 ± 1.1	171 ± 1.7	170 ± 1.2	166 ± 1.9	170 ± 1.4
Heart rate (bpm)	74 ± 1.7	73 ± 1.7	71 ± 2.4	71 ± 2.0	76 ± 2.6	72 ± 1.8
Systolic BP (mmHg)	127 ± 1.6	159 ± 3.0***	128 ± 1.6	164 ± 2.0***	128 ± 1.6	161 ± 2.0***
Diastolic BP (mmHg)	78 ± 1.1	93 ± .77***	75 ± 1.2	95 ± .52***	76 ± 1.5	94 ± 1.5***
PP (mmHg)	49 ± 1.4	66 ± 2.8***	52 ± 1.7	69 ± 1.9***	53 ± 2.5	68 ± 2.2***
Glucose (mmol/l)	5.2 ± .10	5.2 ± 0.14	8.4 ± 0.49^ǂǂǂ^	8.5 ± 0.51^ǂǂǂ^	11 ± 0.72^ǂǂǂ^	9.1 ± 0.43^ǂǂǂ^
Cholesterol (mmol/l)	5.1 ± .24	4.7 ± 0.24	4.1 ± 0.3	4.0 ± 0.16	4.1 ± .22	3.9 ± 0.11
HDL-C (mmol/l)	1.3 ± .07	1.5 ± 0.13	1.2 ± 0.09	1.2 ± .04	1.2 ± 0.12	1.2 ± 0.05
LDL-C (mmol/l)	3.1 ± .23	2.6 ± 0.28	2.2 ± 0.21^ǂ^	1.9 ± 0.11^ǂ^	1.9 ± 0.16^ǂ^	1.8 ± 0.10^ǂ^
Triglycerides (mmol/l)	1.9 ± 1.3	1.6 ± 0.13	2.0 ± 0.34	1.9 ± 0.16	2.4 ± 0.33	2.4 ± 0.20
History anti-hyperglycaemic and anti-hypertensive medication (%)						
Biguanides	8.62	1.41	26.83^ǂǂ^	47.30^ǂǂ^	47.30^ǂǂǂ^	50.72^ǂǂ^
DPP-4 inhibitors	0	0	4.88^ǂǂ^	6.76^ǂǂ^	0%	7.46^ǂǂ^
Sulphonylureas	0	0	17.07^ǂǂ^	41.89^ǂǂ^	33.33^ǂǂ^	41.79^ǂǂ^
Insulin	0	0	24.39^ǂǂ^	16.22^ǂǂ^	40.48^ǂǂ^	25.37^ǂǂ^
ACE inhibitors	0	16.90***	0	33.78***	0	46.27***
Ang II antagonists	0	15.14***	0	20.27***	0	19.40***
Beta-blockers	0	18.31***	0	49.32***	0	58.21***
Ca^2^+ channel blockers	0	11.27***	0	14.86***	0	11.94***

*** p < 0.001 vs same group, different condition, ^ǂ^ p < 0.05, ^ǂǂ^ p < 0.01, ^ǂǂǂ^ p < 0.001 vs obese group, same condition, ^×××^ p < 0.001 vs T2D group, same condition

### The co-existence of obesity and T2D in Normotensive patients had additive effects on the prevalence of LVH

The presence of LVH was determined by assessing the clinical hypertrophy indices LVPWd and LV mass, which were derived from M-Mode measurements (Table 2). Additional M-Mode measures are shown in Additional file 1: Table S1.

**Table 2:** Echocardiography measurements of left ventricular structure

Group	Obese		T2D		Obese/T2D	
Condition	Norm.	Hyper.	Norm.	Hyper.	Norm.	Hyper.
M-mode measurements						
IVSd (cm)	1.0 ± 0.03	1.1 ± 0.02**	1.1 ± 0.03	1.1 ± 0.02	1.1 ± 0.03	1.3 ± 0.02^ǂǂǂ,×××^
LVIDd (cm)	4.9 ± 0.08	4.8 ± 0.08	4.7 ± 0.11	4.6 ± 0.08	4.6 ± 0.10	4.8 ± 0.05
LVPWd (cm)	0.9 ± 02	1.1 ± 0.03	1.0 ± 0.03^ǂ^	1.1 ± 0.03	1.1 ± 0.03^ǂǂǂ^	1.2 ± 0.02***^,ǂǂǂ,×××^
LV mass (g)	159 ± 5.7	191 ± 8.2**	172 ± 8.0	187 ± 5.1	191 ± 8.2	233 ± 6.2***^, ǂǂǂ,×××^
LV mass/BSA	76 ± 2.4	88 ± 3.5	90 ± 3.9	103 ± 4.3	90 ± 4.3	106 ± 4.0^ǂǂ^

** p < 0.01, *** p < 0.001, vs same group, different condition; ^ǂ^ p < 0.05, ^ǂǂ^ p < 0.01, ^ǂǂǂ^ p < 0.001, vs obese group, same condition; ^×××^ p < 0.001 vs T2D group, same condition

We first confirmed that our patient population exhibited normal associations between age and BMI with indices of LVH (Additional file 1: Table S2). Age independently correlated with LVPWd (r^2^ = 0.35, p < 0.001), LV mass (r^2^ = 0.24, p < 0.01), LV mass/BSA (g/m^2^) (r^2^ = 0.34, p < 0.001), LV mass/height (g/m^2.7^) (r^2^ = 0.26, p < 0.01) and RWT (r^2^ = 0.38, p < 0.001) in both normotensive and hypertensive subjects. BMI independently correlated with LVPWd (r^2^ = 0.15, p < 0.05), LV mass (r^2^ = 0.16, p < 0.05) and LV mass/height (g/m^2.7^; r^2^ = 0.39, p < 0.001). The same was true for hypertensive patients with the addition of a correlation between BMI and LV mass/BSA (g/m^2^; r^2^ = −0.25, p = 001; Additional file 1: Table S2).

LVPWd was increased in H.Obese and N.Obese/T2D groups vs N.Obese (1.1 ± 0.03 and 1.1 ± 0.03, vs 0.9 ± 02, p < 0.05 and p < 0.001 respectively) and in H.Obese/T2D patients vs all other groups (1.2 ± 0.02, p < 0.001, Table 2). The prevalence of patients within each group exhibiting LVPWd above recommended ASE guidelines (0.9 cm females, 1.0 cm males) were increase between N.Obese vs H.Obese (35.71 vs 64.71%, p < 0.05), N.T2D vs H.T2D (39.39 vs 63.89%, p < 0.05), N.Obese/T2D vs H.Obese/T2D (64.10 vs 92.54%, p < 0.05) and in both normotensive and hypertensive Obese/T2D groups in comparison to Obese and T2D groups alone (p < 0.05). This shows that in the absence of hypertension, LVPWd measures that are indicative of LVH are present in obese and T2D patients (albeit at a lower prevalence than in hypertensive patients), and that the co-existence of these stresses had an additive effect on the prevalence of LVPWd above ASE guidelines.

**Fig. 1:**
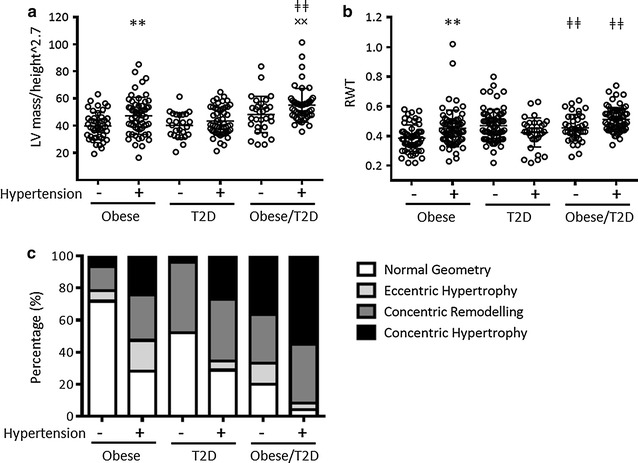
**a** LV mass/height (g/m^2.7^), **b** RWT, *Error bars* represented as mean ± SD. **c** Percentage of subjects with normal LV geometry, eccentric hypertrophy, concentric remodelling or concentric hypertrophy. **p < 0.01, vs same group, different condition; ^ǂǂ^p < 0.01, vs obese group, same condition; ^××^p < 0.01, vs T2D group, same condition

Estimated LV mass (grams) was increased between N.Obese vs H.Obese (159 ± 5.7 vs 191 ± 8.2, p < 0.01), N.Obese/T2D vs H.Obese/T2D (191 ± 8.2 vs 233 ± 6.2, p < 0.001) and in both H.Obese and H.T2D groups vs H.Obese/T2D (191 ± 8.2 and 187 ± 5.1 vs 233 ± 6.2, both p < 0.001). When indexed to BSA (g/m^2^), LV mass remained increased between only H.Obese vs H.Obese/T2D (88 ± 3.5 vs 106 ± 4.0, p < 0.01, Table 2). However, as indexation of LV mass to BSA in obese patients has been suggested to be inaccurate [13], we also indexed to height^2.7. Using this method, LV mass/height (g/m^2.7^) was increased in H.Obese vs N.Obese (47 ± 1.8 vs 40 ± 1.5, p < 0.01), N.Obese/T2D vs N.Obese (48 ± 2.4 vs 40 ± 1.5, p < 0.05) and between H.Obese/T2D vs both H.Obese and H.T2D (55 ± 1.8 vs 47 ± 1.8 and 43 ± 1.4, p < 0.01 and p < 0.001 respectively) (Fig. 1a). Alarmingly, LV mass/height was comparable between normotensive vs hypertensive T2D and Obese/T2D groups (p = 1.0 and p = 0.178, respectively, by ANOVA). When considering normotensive vs hypertensive Obese/T2D groups independently via a student’s *t* test, a significant difference in LV mass/height was observed (p = 0.023, Fig. 1a).

### No difference in the prevalence of concentric remodelling and concentric hypertrophy was observed between normotensive and hypertensive obese/T2D patients

To determine whether the observed increases in LVPWd and LV mass were associated with alterations in LV geometry, RWT was calculated and used with LV mass/height to identify normal LV geometry, EH, CR or CH [12]. Hypertension increased RWT in obese patients (p < 0.01), while the coexistence of obesity and T2D increased RWT compared with obesity alone in both normotensive and hypertensive patients (p < 0.01, Fig. 1b). Calculated LV geometry patterns are shown in Fig. 1c and Additional file 1: Figure S1. The percentage of patients with normal LV geometry decreased between N.Obese vs H.Obese (71 vs 28%, p < 0.001), N.T2D vs H.T2D (52 vs 29%, p < 0.05), N.Obese/T2D vs H.Obese/T2D (20 vs 4%, p < 0.05), N.Obese vs N.Obese/T2D (p < 0.001) and H.Obese vs H.Obese/T2D (p < 0.01). The percentage of patients with EH increased between N.Obese vs H.Obese (6.5 vs 19%, p < 0.05) and decreased between H.Obese vs both H.T2D and H.Obese/T2D (19 vs 4.8% and 4% respectively, p < 0.05). The percentage of patients with CR increased between N.Obese vs N.T2D (15 vs 44%, p < 0.01). The percentage of patients with CH increased between N.Obese vs H.Obese (6 vs 24%, p < 0.01), N.T2D vs H.T2D (4 vs 27%, p < 0.01), both N.Obese and N.T2D vs N.Obese/T2D (6 and 4% vs 37%, p < 0.001) and both H.Obese and H.T2D vs H.Obese/T2D (24 and 27% vs 55%, p < 0.001). As with LV mass/height, the prevalence of CR and CH were comparable between normotensive vs hypertensive Obese/T2D groups (p = 0.629 and p = 0.164 respectively). This suggests that, in the absence of hypertension, obesity and T2D have an additive effect on the development of CH.

### Assessment of unadjusted LVPWd alone underestimates LV remodelling in normotensive obese and T2D patients

**Fig. 2:**
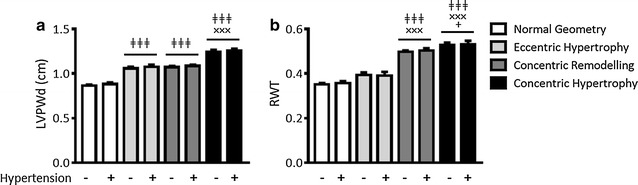
Normotensive and hypertensive patients grouped based on characterisation of normal LV geometry, eccentric hypertrophy, concentric remodelling and concentric hypertrophy. **a** LVPWd (cm), **b** RWT. Data represented as mean ± SEM. ^ǂǂǂ^p < 0.001 vs normal geometry, ^×××^p < 0.001 vs eccentric hypertrophy, ^+^p < 0.05 vs concentric remodelling

Due to discrepancies in past studies that have assessed LVH in normotensive patients with metabolic syndrome [14], we determined whether different outcomes in assessment of LVH would be obtained using unadjusted LVPWd, which is commonly used in clinical practice, vs RWT. Interestingly, in those patients across all groups characterised with CR, 44% of normotensive patients and 25% hypertensive patients (p < 0.05) exhibited LVPWd within normal ASE ranges. This analysis was determined using the preferred formula ((IVSd + LVPWd)/LVID) to derive RWT, as this formula assumes asymmetric LV remodelling by taking into account both septal and posterior aspects of the LV chamber. When using an alternative formula that does not include IVSd ((2 × LVPWd)/LVIDd) similar results were obtained (data not shown). These findings are further supported by the observed differences in LVPWd between LV geometric patterns, with patients characterised with EH and CR exhibiting comparable LVPWd (1.07 ± 0.01 vs 1.07 ± 0.01), greater than that detected in patients with normal LV geometry (0.83 ± 0.01, p < 0.001) and lower than that detected in patients with CH (1.25 ± 0.02, p < 0.001, Fig. 2a). As expected due to LV geometric characterisation criteria, RWT was comparable between patients with normal LV geometry and EH (0.35 ± 0.01 vs 0.39 ± 0.01), increased in those exhibiting CR (0.49 ± 0.01, p < 0.001 vs normal and EH) and interestingly was further increased in those with CH (0.53 ± 0.02, p < 0.001 vs normal and EH, p < 0.05 vs CR, Fig. 2b). These results suggest that in obese and/or T2D patients, assessment of unadjusted LVPWd alone may underestimate the presence of LV remodelling and as such, the additional use of RWT may provide a more sensitive measure in these patients.

### LV geometric patterns associate with differing grades of diastolic dysfunction

To assess the prognostic value of characterising LV remodelling in normotensive obese and/or T2D patients, multivariable logistic regression analysis was used to determine whether LV geometric patterns predicted diastolic dysfunction.

Again we first confirmed that our patient population exhibited normal associations, in this case between age and indices of diastolic function (Additional file 1: Table S2). In normotensive patients, age correlated with E/A ratio (r = −0.25, p < 0.001), E/E′ (r = 0.23, p < 0.01), DT (r = 0.11, p < 0.05) and LAVi (r = 0.28, p < 0.001). The same was true for hypertensive patients, with the exception of LAVi. Associations between BMI and indices of diastolic function were also assessed, as previous studies have yielded conflicting results in this area [15, 16]. However, no associations between BMI with indices of diastolic function were observed (Additional file 1: Table S2).

CR was a predictor of both grade 1 DD (odds ratio (OR) 3.487, p = 0.038) and grade 3 DD (OR 2.157, p = 0.029) when including sex and BMI as covariates. With the addition of age as a covariate, the association with grade 1 DD remained (OR 3.474, p = 0.045), and was lost with grade 3 DD (OR 1.9, p = 0.071). CH proved to be a stronger predictor of grade 3 DD (OR 3.7, p < 0.001) with the inclusion of sex and BMI as covariates. This association was only slightly attenuated with the addition of age as a covariate (OR 3.2, p < 0.005). Supporting these findings, RWT and LV mass/height were identified as predictors of grade 3 DD (OR 30.28, p < 0.001 and OR 1.051, p < 0.001 respectively) when including sex and BMI as covariates. With the addition of age as covariate, the association between LV mass/height with grade 3 DD was lost (OR 1.011, p = 0.337) and was moderately attenuated in relation to RWT (OR 19.245, p = 0.012; Table 3). These data suggest, that the presence of CR is a predictor of grade 1 DD and CH and RWT are predictors of grade 3 DD, independent of sex, age and BMI in obese and/or T2D patients.

**Table 3:** Association between left ventricular geometry and structure with diastolic dysfunction

LVH type/indices	Grade 1 DD		Grade 2 DD		Grade 3 DD	
	OR (95% CI)	p value	OR (95% CI)	p value	OR (95% CI)	p value
Covariates: LVH type/indices, sex and BMI						
EH	3.1 (0.2–55.7)	N/S	2.5 (0.7–8.6)	N/S	0.6 (0.2–2.1)	N/S
CR	3.5 (0.1–11.3)	0.038	1.1 (0.5–2.1)	N/S	2.2 (1.1–4.3)	0.029
CH	0.5 (0.1–3.0)	N/S	0.7 (0.3–1.4)	N/S	3.7 (1.7–8.0)	0.000
RWT	11.1 (0.2–562)	N/S	0.2 (0.02–2.6)	N/S	30.1 (3.2–286)	0.000
LV mass/height	1.1 (1.0–1.1)	N/S	1.0 (0.9–1.0)	N/S	1.1 (1.0–1.1)	0.000
Covariates: LVH type/indices, sex, BMI and age						
EH	2.4 (0.1–46.1)	N/S	2.8 (0.8–10.2)	N/S	0.8 (0.2–3.2)	N/S
CR	3.5 (1.1–11.7)	0.045	1.2 (0.6–2.4)	N/S	1.9 (0.9–3.9)	0.071
CH	0.7 (0.–4.8)	N/S	0.8 (0.4–1.7)	N/S	3.2 (1.4–7.2)	0.003
RWT	1.0 (0.9–1.0)	N/S	1.0 (0.9–1.0)	N/S	19.2 (1.9–193)	0.012
LV mass/height	1.1 (1.0–1.1)	N/S	1.0 (0.9–1.1)	N/S	1.0 (1.0–1.0)	0.337

### The co-existence of obesity and t2D in normotensive patients had an additive effect on the prevalence of grade 3 diastolic dysfunction

**Fig. 3:**
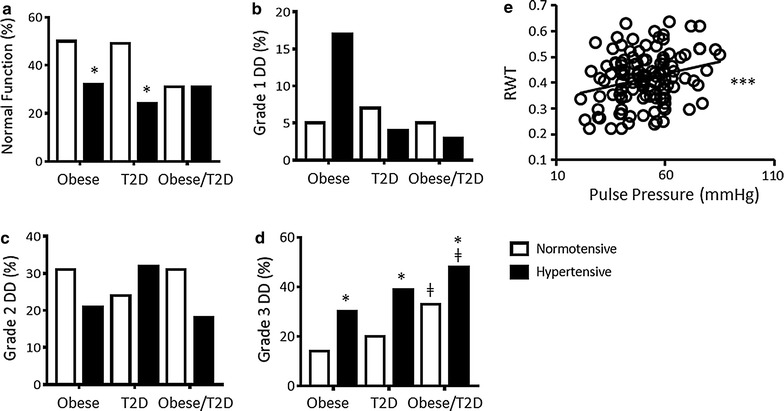
Percentage of normotensive and hypertensive obese, T2D and obese/T2D groups with **a** normal diastolic function, **b** grade 1 DD, **c** grade 2 DD and **d** grade 3 DD. **e** Linear regression analysis between RWT and Pulse Pressure (mmHg) in normotensive obese and/or T2D patients, ***p < 0.001 via linear regression analysis. Accounting for sex and age; *p < 0.05 vs same group, different condition, ^ǂ^p < 0.05, ^ǂǂ^p < 0.01, ^ǂǂǂ^p < 0.001 vs obese group, same condition, ^×××^p < 0.001 vs T2D group, same condition

The prevalence of DD in normotensive vs hypertensive obese and/or T2D groups irrespective of LV geometric type was determined (Fig. 3a–d). Accounting for sex and age, normal diastolic function decreased between N.Obese vs H.Obese (50 vs 32.4%, p < 0.05) and N.T2D vs H.T2D (48.8 vs 24.3%, p < 0.05) groups, grade 1 DD increased between N.Obese vs H.Obese (5 vs 17%, p < 0.05) and grade 3 DD increased between N.Obese vs H.Obese (13.8 vs 39.2%, p < 0.05), N.T2D vs H.T2D (19.5 vs 47.8%, p < 0.05) and N.Obese/T2D vs H.Obese/T2D (34.1% vs 47.8, p < 0.05) groups. The prevalence of Grade 3 DD in both normotensive and hypertensive Obese/T2D patients was greater than that detected in obese (p < 0.01) and T2D (p < 0.05) groups alone. These results suggest that there is an additive effect on diastolic decline when obesity and T2D co-exist, compared to when these stresses present individually.

Systolic function was also assessed, however, all groups exhibited indices within the range of normal, as per ASE guidelines (Additional file 1: Table S1).

### Pulse pressure is an independent predictor of increased RWT in normotensive patients

In order to determine which normotensive obese and T2D patients were at greatest risk of developing LVH, linear regression analysis was used to determine whether routinely measured metabolic parameters (Table 1) associated with RWT. RWT was chosen as the independent variable to detect risk of LVH, as RWT provided the most sensitive measure to detect LVH in our normotensive patients. In addition, concentric remodelling and concentric hypertrophy were the most prevalent types of LV geometry detected, for which both have the common requirement for increased RWT.

Pulse pressure (Fig. 3e) and fasting glucose (Additional file 1: Figure S3A) were associated with RWT (r^2^ = 0.28, p < 0.001 and r^2^ = 0.33, p < 0.001, respectively). Specifically, pulse pressure ≥54 mmHg and fasting glucose ≥7.7 mmol/l were associated with a RWT > 42, a value characteristic of concentric remodelling and also concentric hypertrophy when accompanied by an increase in LV mass. When accounting for sex, age and BMI, the correlations between pulse pressure (r^2^ = 0.33, p < 0.001), fasting glucose (r^2^ = 0.35, p < 0.001) and RWT were slightly strengthened. This suggests that these parameters can be used to predict risk in normotensive obese and/or T2D patients, independent of age and BMI. The same associations were not detected in hypertensive obese and/or T2D patients (Additional file 1: Figure S3B, C). Presumably due to the stronger influences of hypertension on cardiac structure in comparison to obesity and T2D.

### Normotensive metabolically non-healthy obese patients exhibited increased prevalence of concentric remodelling and diastolic dysfunction

**Fig. 4:**
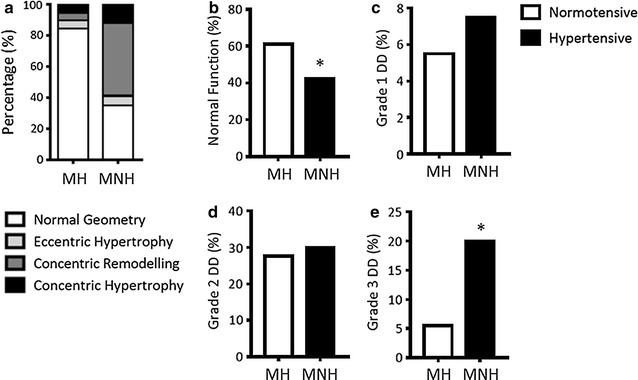
Percentage of metabolically healthy (MH) and metabolically non-healthy (MNH) normotensive with **a** normal LV geometry, eccentric hypertrophy, concentric remodelling and concentric hypertrophy, **b** normal diastolic function, **c** grade 1 DD, **d** grade 2 DD, and **e** grade 3 DD. Accounting for sex and age; *p < 0.05y vs MH

As BMI did not associate with RWT or indices of DD in our patients (Additional file 1: Tables S2, S3), we aimed to determine what factors in normotensive obese patients were associated with the observed alterations in LV geometry and DD (Figs. 1c, 3b–d). To do this, N.Obese patients were stratified into metabolically healthy and metabolically non-healthy N.Obese groups (see “Methods” section). In doing so, we determined that metabolically healthy N.Obese patients have a relatively low risk of concentric remodelling and concentric hypertrophy compared with metabolically non-healthy N.Obese, exhibiting normal LV geometry at a prevalence of 84.8 vs 35% (p < 0.001), concentric remodelling at 5 vs 47% (p < 0.001) and concentric hypertrophy at 5 vs 12% (p < 0.05, Fig. 4a). The prevalence of eccentric remodelling remained low in both metabolically healthy and non-healthy N.Obese patients (5.2 vs 6%, Fig. 4a). In addition, the prevalence of concentric remodelling and concentric hypertrophy in normotensive metabolically non-health obese patients was comparable to H.Obese patients (47 and 12% vs 47 and 25%, p = 1.0 and p = 0.13 respectively), suggesting that in obese patients, metabolic abnormalities have effects similar to hypertension on LV remodelling. The prevalence of normal diastolic function declined between metabolically healthy N.Obese vs metabolically non-healthy N.Obese (61 vs 42%, p < 0.05, Fig. 4b), while grade 3 DD increased (5.5 vs 20%, p < 0.05, Fig. 4e). There were no differences in grade 1 and 2 DD between groups (Fig. 4c, d).

## Discussion

Despite professional knowledge of the risks associated with obesity and T2D in the development of cardiovascular disease, monitoring blood pressure remains the first line method when assessing risk in normotensive obese and/or T2D patients. While this practice allows for the early detection of hypertension, it may not be sufficient to allow for the early detection of LVH and DD. Indeed, results from this current study suggest that in the absence of hypertension, LVH and DD may be present and detectable via TTE in normotensive obese and/or T2D patients. The major findings from this study are that; (1) Significant alterations in LV remodelling indicative of LVH were detected in normotensive metabolically non-healthy obese, T2D and obese/T2D patients. (2) Assessment of LVPWd via recommended ASE guidelines underestimated the presence of LV remodelling. (3) Concentric remodelling was a predictor of grade 1 DD and concentric hypertrophy and RWT predictors of grade 3 DD, independent of sex, age and BMI. (4) Normotensive patients with an increased risk of a RWT > 42 with those exhibiting pulse pressure ≥54 mmHg.

Considering obese patients first, metabolically healthy N.Obese patients exhibited a relatively low prevalence of LV remodelling, with >80% of these patients exhibiting normal LV geometry. It was only in those N.Obese patients characterised with “poor” metabolic health that significant alterations in LV geometry were detected. This was particularly true for concentric remodelling (47%), which was 9.4 times higher than that in metabolically healthy N.Obese patients and 1.6 times higher than that in H.Obese patients. This suggests that obesity per se, as defined by a BMI ≥ 30 (kg/m^2^), does not promote the development of concentric remodelling, but rather it is the metabolic health status of the patient with a BMI ≥ 30 (kg/m^2^) that has the greatest influence on increases in RWT. Supporting this claim, our study showed that BMI had no association with RWT or diagnosis of concentric remodelling. Therefore, these results suggest additional risk assessment should be performed in normotensive obese patients with more than one of the following metabolic parameters; fasting glucose >5.5 mmol/l, HDL-C <1.4 mmol/l, LDL-C >2.6 mmol/l, cholesterol >5.5 mmol/l and triglycerides >1.8 mmol.

H.Obese patients on the other hand exhibited not only concentric remodelling (28%) but also concentric hypertrophy (25%), at a prevalence 4.8 and 2 times higher than that in metabolically healthy and non-healthy N.Obese patients respectively. This is consistent with previous studies in which autopsy results identified hypertensive obese patients to exhibit both concentric remodelling and concentric hypertrophy and for normotensive obese patients to present with mainly concentric remodelling [17]. N.T2D patients exhibited similar alterations in LV geometry as metabolically non-healthy obese, showing a comparable prevalence of concentric remodelling (44%) and a low prevalence of concentric hypertrophy (4%), 6.75 times lower than that in H.T2D patients. Interestingly, it was only in those N.Obese/T2D patients in which the predominant LV geometric patterns detected included both concentric remodelling (30%) and concentric hypertrophy (36%), which was comparable to that in H.Obese patients. These results suggests that the co-existence of obesity and T2D in normotensive patients had an effect on the development of CH which was similar to the negative influence hypertension had when coupled with obesity or T2D alone.

To our knowledge, this is the first study reporting that the use of unadjusted LVPWd vs RWT underestimated the presence of LV remodelling in obese and T2D patients. This had particular relevance in normotensive patients, with 45% of those characterised with concentric remodelling exhibiting LVPWd within the ranges of normal ASE guidelines. This result was unchanged regardless of how RWT was derived. However, consideration of septal wall thickening was preferred due to the presence of asymmetric LV remodelling in some patients, with IVSd showing similar patterns of enlargement as LVPWd within our groups (Additional file 1: Table S1). Of note, this increase in septal thickening was accompanied by aortic stenosis, with a thinning of the proximal ascending aorta correlating with increased IVSd (r = 0.24, p = 0.001, data not shown), a correlation that has previously been identified in subjects with asymmetric remodelling [18]. These data suggest that in normotensive obese and/or T2D patients assessment of unadjusted LVPWd alone may underestimate the presence of LV remodelling (both symmetric and asymmetric) and, as such, the additional use of RWT may provide a more sensitive measure in these patients.

The implications of underestimating LV remodelling are evident in this study with the use of multivariable logistic regression analysis identifying an association between concentric remodelling with diagnosis of grade 1 DD and concentric hypertrophy and RWT with grade 3 DD (independent of sex, age and BMI). This suggests that reliance on unadjusted LVPWd in normotensive obese and T2D patients underestimates not only LV remodelling, but also associated cardiovascular risk. While previous studies have identified associations between LV geometry with adverse cardiac function and cardiovascular risk [19–24], these studies have predominately focused on hypertensive patients or have not had access to subject data regarding hypertension. The data from this study provides insight into the prognostic value of LV geometry, specifically in normotensive obese and T2D patients.

While subject numbers in the present study were low, the identification that pulse pressure and fasting glucose to be associated with RWT may assist health practitioners in narrowing down the otherwise large patient pool of at risk individuals to which this study relates, with pulse pressure ≥54 mmHg and fasting glucose ≥7.7 mmol predictors of RWT > 42. RWT was chosen as an independent variable to detect risk of LVH due to concentric remodelling and concentric hypertrophy being the predominant LV geometric patterns detected for which both have the common requirement for increased RWT. In addition, RWT provided a more sensitive measure to detected LV remodelling in our patients and increases in RWT have previously been associated with adverse cardiovascular events [9, 25, 26]. In healthy subjects normal pulse pressure levels have been detected at 40 mmHg. The recorded pulse pressure measurements in the current study were increased, averaging >50 mmHg in normotensive and >60 mmHg in hypertensive patients. Similar increases in pulse pressure have previously been recorded in subjects with metabolic syndrome [27, 28]. To our knowledge, this is the first study identifying the use of pulse pressure as a predictor of increased RWT that can be applied to all normotensive obese, T2D and obese/T2D patients. In a dataset from the HyperGEN study [29] normotensive subjects with a higher pulse pressure (>60 mmHg) were associated with an increased prevalence of thickening of the LVPWd and increased RWT. This pulse pressure quartile however contained a lower average BMI (27.06 kg/m^2^) and percentage of T2D subjects (3.97%) compared to current study.

Fasting glucose, has been associated with indices of LVH in the past [30]. Furthermore, patients with impaired fasting glucose or T2D have previously been reported to exhibit a 9 and 11% respectively increase in the prevalence in concentric remodelling than that in euglycemic patients (of note, 46% of patients were hypertensive) [31]. The identification of the association with fasting glucose with RWT in the current study was strengthened when accounting for age and BMI. When comparing the use of fasting glucose vs pulse pressure as a predictor of increased RWT via multivariable stepwise regression analysis, fasting glucose was a stronger predictor of RWT (data not shown). In regards to the practicality of using pulse pressure and fasting glucose as markers of increased risk, these parameters are already routinely collected in normotensive obese and T2D patients, therefore their use will not result in an additional burden in either cost or time. However, it should be noted that although the presence of elevated pulse pressure and fasting glucose were associated with LV remodelling, this study is not suggesting that these are a requirement and, as such should not provide a means to exclude patients at risk.

There are some limitations that need to be considered when interpreting the findings from this study. In regards to diastolic function, the reader is reminded that these measurements were obtained from an assessment at one time point and that diastolic function may vary between examinations. Furthermore, the size of the research groups were limited, more hypertensive vs normotensive patients were included in the study and analysis was restricted to patients of one ethnic group. The strengths of the study included providing clearly separated normotensive and hypertensive obese, T2D and obese/T2D patients. Allowing us to tease out the influences of these conditions individually and in combination. Furthermore, despite the restricted patient numbers in the study we ensured that our dataset exhibited the normal associations between age with diastolic decline and LV structure, adding confidence that the findings from this study are relevant to the wider population. In addition, we have focused our analysis on echocardiography techniques and clinical measurements that are already routinely measured in day to day practice, increasing the application potential of the study.

## Conclusion

In summary, the data from this current study suggest that in the absence of hypertension, metabolically non-healthy obese, T2D and obese/T2D patients are at risk of LVH and LV remodelling and that this risk is increased when accompanied by increased pulse pressure or fasting glucose. The identification of LVPWd vs RWT to underestimate LV remodelling in normotensive obese and/or T2D patients suggests that the use of RWT may provide a more sensitive measure in future studies and in risk assessment in these patient groups.

## Additional file

**Additional file 1.** Additional tables and figures.

## Abbreviations

ASE: American Society of Echocardiography
BMI: body mass index
BP: blood pressure
BSA: body surface area
BW: body weight
CH: concentric hypertrophy
CR: concentric remodelling
DD: diastolic dysfunction
DT: deceleration times
EH: eccentric hypertrophy
HDL-C: high density lipoprotein cholesterol
IVSd: interventricular septum dimension
LDL-C: low density lipoprotein cholesterol
LV: left ventricle
LVH: left ventricular hypertrophy
LVIDd: left ventricular internal diastolic dimension
LVIDs: left ventricular internal systolic dimension
LVMI: left ventricular mass index
LVPWd: left ventricular posterior wall diameter
RWT: relative wall thickness
T2D: type 2 diabetes
