# Erratum to: Obesity and type 2 diabetes have additive effects on left ventricular remodelling in normotensive patients-a cross sectional study

**DOI:** 10.1186/s12933-017-0535-5

**Published:** 2017-04-20

**Authors:** Kirstie A. De Jong, Juliane K. Czeczor, Smithamol Sithara, Kevin McEwen, Gary D. Lopaschuk, Alan Appelbe, Kimberly Cukier, Mark Kotowicz, Sean L. McGee

**Affiliations:** 1 Metabolic Research Unit, Metabolic Reprogramming Laboratory, School of Medicine, Deakin University, Waurn Ponds, VIC Australia; 2 Institute for Clinical Diabetology, German Diabetes Center, Leibniz Center for Diabetes Research, Heinrich-Heine University, c/o Auf’m Hennekamp 65, 40225 Düsseldorf, Germany; 3 German Center of Diabetes Research, Ingolstädter Landstraße 1, 85764 München-Neuherberg, Germany; 4 Department of Pediatrics, University of Alberta, Edmonton, AB T6G 2H7 Canada; 5 Department of Pharmacology, University of Alberta, Edmonton, AB T6G 2H7 Canada; 6 Cardiology Department, Barwon Health, University Hospital Geelong, Victoria, Australia; 7 Geelong Endocrinology and Diabetes Centre, Geelong, VIC Australia; 8 Endocrinology Department, Barwon Health, University Hospital, Geelong, VIC Australia; 9 School of Medicine, Deakin University, Waurn Ponds, VIC Australia; 10 Melbourne Medical School-Western Precinct, The University of Melbourne, Victoria, Australia

## Erratum to: Cardiovasc Diabetol (2017) 16:21 DOI 10.1186/s12933-017-0504-z

After publication of the original article [1], it came to the authors’ attention that in the author list Gary Lopaschuk’s surname originally contained a typo. The error has now been rectified in the original article.
