# Supplementary material for: Obesity and type 2 diabetes have additive effects on left ventricular remodelling in normotensive patients-a cross sectional study
Source: Cardiovasc Diabetol. 2017 Feb 8;16:21. doi: 10.1186/s12933-017-0504-z (PMC5299776; doi:10.1186/s12933-017-0504-z)
Supplement: Supplementary file 1 — Additional file 1. Additional tables and figures. [file 12933_2017_504_MOESM1_ESM.docx]

**SUPPLEMENTARY DATA**

**Additional study design information**

The study was conducted using pre-recorded endocrinology and echocardiography data from two separate databases. The first database searched contained basic patient clinical characteristics obtained from the Endocrinology department of the Geelong hospital and the Geelong Endocrinology and Diabetes practice. This database was used to obtain a list of patients at each clinic who were either obese and non-T2D, T2D and non-obese or obese and T2D. Obesity was characterised by a BMI > 30 and T2D as having three or more elevated fasting glucose level readings (>7 mmol/l) within a 12 month period. After this search 2397 patients met the selection criteria (1158 obese only, 572 T2D only, 667 obese and T2D). Additional data collected for each patient from this database included; age, sex, height, weight, blood pressure, fasting glucose, HbA1c %, LDL-C, HLD-C, total cholesterol and triglycerides, history of anti-hyperglycaemic medication, history of anti-hypertensive medication and history of cardiovascular or systemic disease.

These 2397 patients were then cross referenced with the second separate cardiovascular database to determine whether the patients had undergone an echocardiography within 6 months of collection of the basic clinical data above. For which 503 patients were matched. The patient data collected from the cardiology database was; age, sex, height, weight, reason for referral for echocardiography and echocardiography results. Patients with an unclear echocardiography due to technical reasons (largely caused by presence of obesity), age < 18 years, history of systemic or cardiovascular disease and those with systolic only or diastolic only hypertension or well controlled hypertension were excluded from the study. After this point, 353 patients were left that met the selection criteria (129 obese only, 115 T2D only and 109 obese and T2D). These patients were then further separated into normotensive and hypertensive groups. With hypertension characterised as having both elevated diastolic and systolic blood pressure of ≥ 140/90 mmHg, with or without a history of anti-hypertensive medication. Final groups numbers were; normotensive obese, 58; normotensive T2D, n=41; normotensive obese/T2D, n=42, hypertensive obese, n=71; hypertensive T2D, n=74; and hypertensive obese/T2D, n=67.

Table S1.

| **GROUP** | **OBESE** | | **T2D** | | **OBESE/T2D** | |
| --- | --- | --- | --- | --- | --- | --- |
| **Condition** | **Norm.** | **Hyper.** | **Norm.** | **Hyper.** | **Norm.** | **Hyper.** |
| *Glucose Homeostasis* | | | | | | |
| Hba1c | - | - | 7.1 ± 0.3 | 7.6 ± 0.5 | 8.0 ± 0.3 | 7.7 ± 0.2 |
| *M-Mode Measurements* | | | | | | |
| Aortic Root (cm) | 0.3 ± .06 | 3.2 ± .05 | 3.5 ± .08 ǂǂǂ | 3.4 ± .05 ǂ | 3.3 ± .06 | 3.6 ± .05 ǂǂǂ |
| Ascending Aorta (cm) | 3.7 ± .14 | 3.9 ± .10 | 3.8 ± .13 | 4.0 ± .10 | 3.8 ± .14 | 4.1 ± .11 |
| LA Volume index | 29 ± 1.5 | 31 ± 1.2 | 32 ± 2.1 | 33 ± 1.5 | 28 ± 1.9 | 31 ± 1.8 |
| IVSd (cm) | 1.0 ± .03 | 1.1 ± .02 | 1.1 ± .03 ǂǂǂ | 1.1 ± .02 | 1.1 ± .03 ǂǂǂ | 1.3 ± .02 ǂǂǂ ××× |
| LVIDd (cm) | 4.9 ± .08 | 4.8 ± .08 | 4.7 ± .11 | 4.6 ± .08 | 4.6 ± .10 | 4.8 ± .05 |
| LVIDs (cm) | 3.1 ± .12 | 3.1 ± .10 | 3.1 ± .12 | 3.1 ± .11 | 3.0 ± .11 | 3.1 ± .08 |
| Peak E-Wave | 0.8 ± .02 | 0.8 ± .03 | 0.8 ± .04 | 0.8 ± .03 | 0.9 ± 05 | 0.9 ± .05 |
| Peak A-Wave | 0.7 ± .03 | 0.8 ± .04 | 08 ± .04 | 0.9 ± .03 | 0.8 ± .04 | 0.9 ± .04 |
| E/A Ratio | 1.3 ± .07 | 1.1 ± .06 | 1.1 ± .08 | 1.2 ± .06 | 1.1 ± .07 | 1.1 ± .07 |
| E’ | 8.4 ± .43 | 7.6 ± .31 | 6.6 ± .38 | 5.8 ± .22 | 7.1 ± .31 | 6.0 ± .31 |
| E/E’ | 11 ± .46 | 12 ± .8 | 12 ± 1.3 | 14 ± .60 | 14 ± 1.1 | 16 ± .98 ǂǂǂ |
| DT | 225 ± 10 | 223 ± 6.9 | 221 ± 11 | 227 ± 8.9 | 202 ± 7.4 | 230 ± 11 |
| *Systolic Indices Derived from M-Mode Measurements* | | | | | | |
| Stroke Volume | 72.6 ± 3.6 | 71.3 ± 2.8 | 67.3 ± 4.1 | 64.9 ± 3.0 | 70.4 ± 2.9 | 67.0 ± 2.6 |
| Cardiac Output | 5386 ± 267 | 5094 ± 214 | 4308 ± 273 | 4500 ± 212 | 5269 ± 289 | 4727 ± 208 |
| EF% | 61.9 ± 2.1 | 65.0 ± 1.6 | 62.7 ± 2.2 | 63.3 ± 2.04 | 67.8 ± 2.0 | 62.7 ± 1.9 |
| FS% | 34.0 ± 1.4 | 36.3 ± 1.2 | 34.7 ± 1.6 | 35.7 ± 1.5 | 39.4 ± 2.2 | 35.0 ± 1.3 |

ǂ ǂ ǂ p<0.001 vs obese group, same condition, ××× p<0.001 vs T2D group, same condition

Table S2.

| **VARIABLE** | **AGE** | | | | | **BMI** | | | | | | |
| --- | --- | --- | --- | --- | --- | --- | --- | --- | --- | --- | --- | --- |
| **Condition** | **Norm.** | | **Hyper.** | | | **Norm.** | | | **Hyper.** | | | |
|  | p value | *r^2^* | | p value | *r^2^* | | p value | *r^2^* | | p value | *r^2^* |  |
| *Association with indices of Left Ventricular Hypertrophy* | | | | | | | | | | | |  |
| LVPWd | 0.000 | 0.35 | | N/S | 0.07 | | 0.050 | 0.15 | | 0.005 | 0.18 |  |
| LV mass | 0.003 | 0.24 | | 0.063 | 0.11 | | 0.035 | 0.16 | | 0.001 | 0.21 |  |
| LV mass/BSA | 0.000 | 0.36 | | 0.001 | 0.25 | | N/S | -0.07 | | 0.001 | -0.25 |  |
| LV mass/ height^2.7 | 0.004 | 0.26 | | N/S | 0.06 | | 0.000 | 0.39 | | 0.000 | 0.38 |  |
| RWT | 0.000 | 0.38 | | N/S | 0.09 | | N/S | 0.04 | | N/S | 0.07 |  |
| *Association with indices of Diastolic Dysfunction* | | | | | | | | | | | |  |
| E/A ratio | 0.000 | -0.25 | | 0.04 | -0.09 | | N/S | 0.03 | | N/S | -0.03 |  |
| E/E’ | 0.011 | 0.23 | | 0.001 | 0.16 | | N/S | 0.05 | | N/S | 0.29 |  |
| DT | 0.035 | 0.11 | | 0.026 | 0.14 | | N/S | -0.02 | | N/S | -0.07 |  |
| LAVi | 0.003 | 0.28 | | N/S | 0.03 | | N/S | -0.09 | | N/S | -0.08 |  |


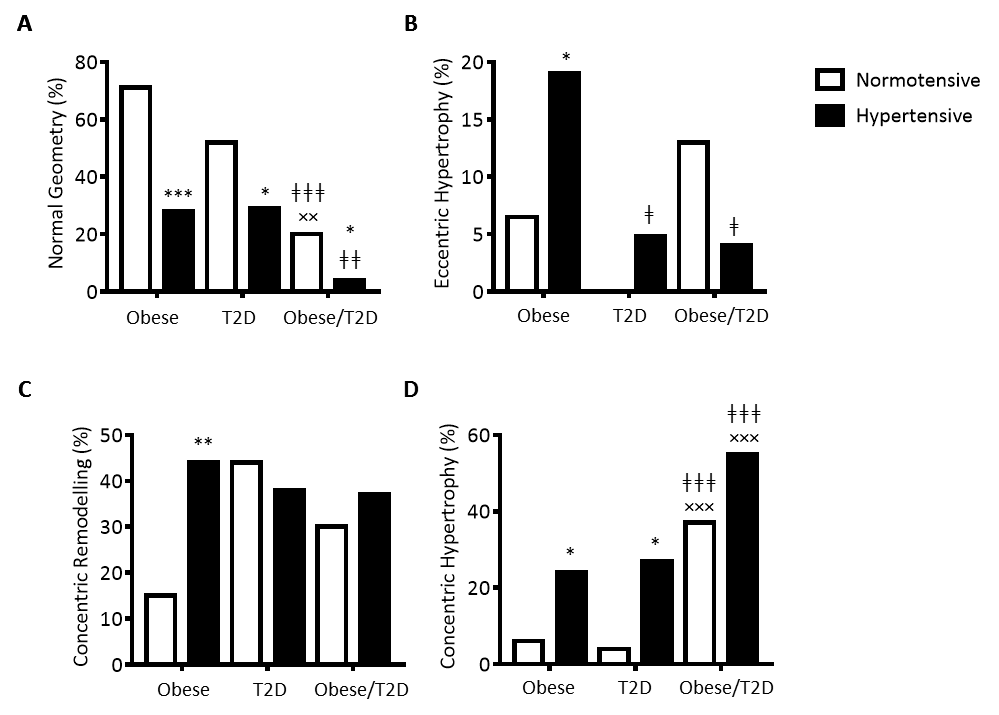


**Figure S1**. Percentage of normotensive and hypertensive obese, T2D and obese/T2D groups with **A** Normal LV geometry, **B** Eccentric hypertrophy, **C** Concentric remodelling and **D** Concentric hypertrophy. * p<0.05, **p<0.01, ***p<0.001 vs same group, different condition, ǂ p<0.05, ǂ ǂ p<0.01, ǂ ǂ ǂ p<0.001 vs obese group, same condition, ××× p<0.001 vs T2D group, same condition.


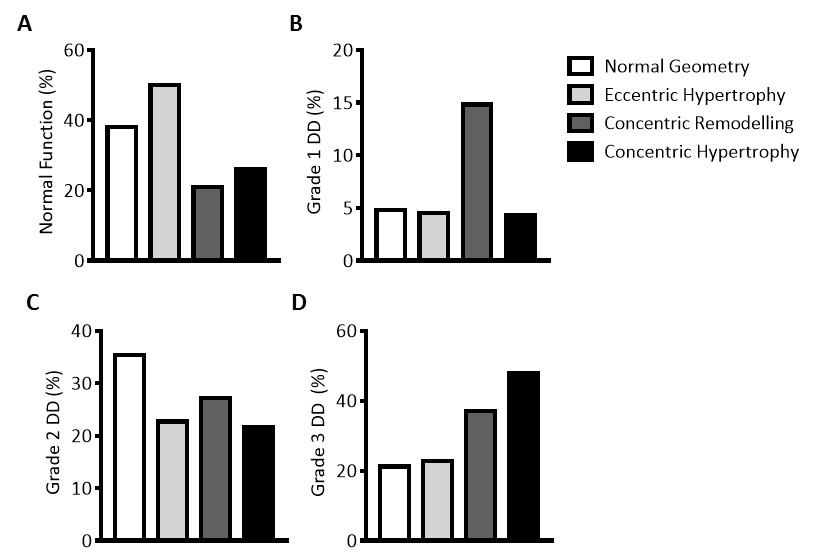


+ǂ

ǂǂ

××

××

ǂ

××

ǂ

×

ǂ

ǂ

×

**Figure S2.** Percentage of patients characterised with normal LV geometry, eccentric hypertrophy, concentric remodelling and concentric hypertrophy with **A** Normal diastolic function, **B** Grade 1 DD, **C** Grade 2 DD and **D** Grade 3 DD. Accounting for sex and age; ǂ p<0.05, ǂ ǂ p<0.01vs normal geometry, × p<0.05, ×× p<0.001 vs eccentric hypertrophy,+ p<0.05 vs concentric remodelling..


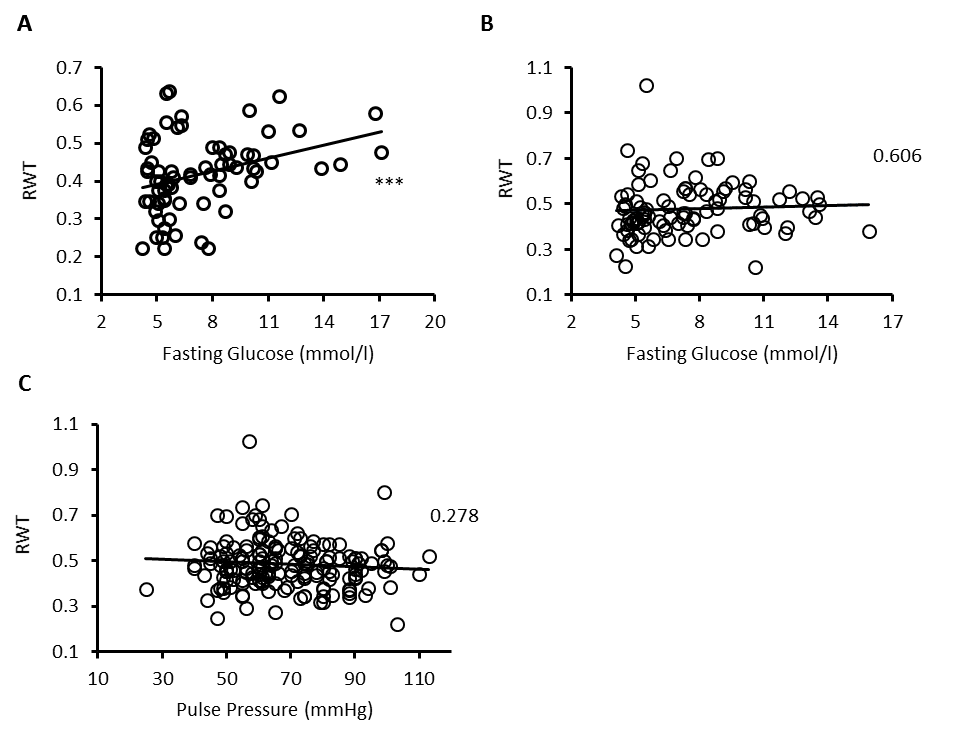


p=0.278

p=0.606

r^2^=0.35 p<0.001

**Figure S3.** Linear regression analysis between RWT and fasting glucose (mmol/l) in obese and/or T2D; **A** normotensive patients and **B** hypertensive patients and **C** linear regression analysis between RWT with pulse Pressure (mmHg) in hypertensive obese and/or T2D patients.
